# Protocol for a constructivist metasynthesis of qualitative research of heroism and paramedic practice

**DOI:** 10.29045/14784726.2021.9.6.2.34

**Published:** 2021-09-01

**Authors:** Nigel Rees, Julia Williams, Chloe Hogan, Lauren Smyth, Thomas Archer

**Affiliations:** Welsh Ambulance Services NHS Trust ORCID iD: https://orcid.org/0000-0001-8799-5335; University of Hertfordshire; Welsh Ambulance Services NHS Trust; Welsh Ambulance Services NHS Trust; Cardiff University

**Keywords:** ambulance, EMS, hero, heroism, paramedic

## Abstract

**Background:**

Exceptional demands have been placed on paramedics and other healthcare workers (HCWs) during the COVID-19 pandemic. An overwhelming outpouring of public support has unfolded, bringing into focus the relationship between paramedics, other HCWs and society, where they are portrayed as heroes. Scholars have studied the notion of heroism to society, and characteristics of such heroic status include: the voluntary nature of a heroic act, risk of physical or social harm, willingness to accept the consequences of action, acting for the benefit of others and without the expectation of gain. While some HCWs and paramedics may reflect these characteristics, many may not. Such heroic narratives can be damaging, stifling meaningful discussion around limits to duties, failing to acknowledge the importance of reciprocity and potentially imposing demands on paramedics and HCWs to be heroic.

**Aim:**

This article prospectively presents the protocol for a metasynthesis which aims to identify, appraise and synthesise the qualitative literature in order to develop theory on heroism and paramedic practice.

**Methods:**

Evolved grounded theory methodology is followed along with the procedural guidelines of Noblit and Hare (1988) to guide the analysis. The Preferred Reporting Items for Systematic Reviews and Meta-analysis Protocols (PRISMA-P) have also been adopted when preparing this protocol and will be followed in the study proper. The protocol has been registered with the International Prospective Register of Systematic Reviews PROSPERO 2021, registration number CRD42021234851.

**Results:**

We do not currently have results, but PRISMA guidelines will be followed when reporting our findings.

**Conclusion:**

Current narratives on heroism and paramedic practice are important in terms of the relationship between paramedics and society. The metasynthesis prospectively reported in this article serves as the first point in our journey of making sense of and developing theory on heroism and paramedic practice.

## Introduction

The COVID-19 pandemic has brought into focus the relationship between society and healthcare workers (HCWs) such as paramedics in an unprecedented way. Exceptional demands have been placed on paramedics and other HCWs globally, which has resulted in an outpouring of public support for them. Across the world, paramedics and other HCWs have been portrayed as heroes; in the UK, public buildings have been lit up in NHS blue, the hashtag #NHSHeroes has trended on social media and a very visible campaign of NHS Heroes been promoted within society by charities, media, government and the wider public (NHS Heroes, 2020).

Scholars have previously studied the notion of heroism to society, and identified that many definitions and differing views exist within the literature. However, risk to self, as well as benefit to others, have been identified as two necessary elements for action to constitute heroism (Kohen et al., 2017; Rankin & Eagly, 2008). Paramedics and many HCWs often experience these elements, especially within the context of a pandemic. However, Franco et al. (2011) suggest that these features are not sufficiently comprehensive, and propose the following characteristics are necessary to meet the criteria of heroic status: the voluntary nature of a heroic act; the risk of potential physical or social harm; a willingness to accept the consequences of the action; acting for the benefit of others; and acting without the expectation of gain. When considered in the light of these characteristics, problems arise with such heroic characterisation for paramedics and many other HCWs. Cox (2020) argues that the heroism narrative within the current COVID-19 pandemic can be damaging, as it stifles meaningful discussion about the limits to healthcare professionals’ duties and treatments, and fails to acknowledge the importance of reciprocity; rather, it can have negative psychological effects on workers themselves through its implication that all healthcare workers have to be heroic.

Authors of this proposal recently conducted a qualitative study and constructed an Evolved Grounded Theory of the tragic choices that paramedics experienced in providing paramedic care during the COVID-19 pandemic (Rees et al 2021). Within this research, we found that while the public held paramedics up as heroes during the COVID-19 pandemic, paramedics in this study were very humble and did not consider themselves to be within this heroic characterisation of them in society, but rather felt they were just doing their job. Tangherlini (2000) found similar but far more cynical and self-deprecatory accounts from paramedics of such heroic status, which countermands the representations from the public and media of them as silent heroes just doing their job. Rather, Tangherlini (2000) found that paramedics tended to present themselves in their stories as anti-heroes quick with a sardonic quip.

Paramedic practice in the UK and internationally has neither medicine’s long history of professional presence nor the occupational research base of other HCWs such as nursing, and it is therefore often assumed to be a hybrid of knowledge and skills taken from other pre-established occupations (Campeau, 2008). Theory is however essential for the development of professional knowledge, and important in defining professional identity. Marrs and Lowry (2006) argue that nursing’s sustained efforts are at least in part intended to define its professional identity. Through this metasynthesis we aim to identify, appraise and synthesise the qualitative literature in order to develop theory on heroism and paramedic practice.

## Methods

This metasynthesis is to be conducted within a constructivist paradigm of inquiry, and it is the epistemological basis of the study which sees the world as constructed, interpreted and experienced by people in their interactions with each other and with wider social systems (Lincoln & Guba, 2000). We draw on the evolved grounded theory methodology (GTM) of Strauss and Corbin (1998) which reflects early social sciences approaches to meta-ethnography and relies on conceptual coding and construction of new theory (Timulak et al., 2009). We also use Noblit and Hare’s (1988) procedural guidelines to guide the analysis: a method originally developed for meta-ethnographic studies, but also intended to guide other interpretive works, and frequently now used in metasyntheses (Bondas & Hall, 2007a, 2007b; Nolte et al., 2017). Members of our study team have used these methodologies previously to construct metasyntheses of the literature on paramedic and emergency care staff (Rees et al., 2015). Noblit and Hare’s (1988) procedural guidelines include the following activities:

- Getting started – deciding what the study is going to be about and the aim.
- Deciding what is relevant to the initial interest;
- Reading the studies repeatedly, analysing and noting interpretive metaphors (themes, concepts);
- Determining how the studies are related, listing key metaphors in each study and their relationship to each other;
- Translating by reading each article several times, searching for metaphors, concepts or categories across data;
- Synthesising the findings to create a new whole of the parts by juxtaposing metaphors (themes), concepts or categories and relationships that refute emerging patterns, noting discordance, dissonance and overlap; and
- Expressing the synthesis in written form.

### Getting started (the search)

Searches will be undertaken of the databases CINAHL®, MEDLINE®, OVID ®, SSCI – Social Sciences Citation Index (via the Web of Science), Scopus ® and Psych INFO®. Our search strategy will include the search terms of (‘Hero’ OR ‘Heroic’ OR ‘Heroism’ OR ‘Heroine’) AND (‘Paramedic’ OR ‘Emergency carer’ OR ‘EMT’ OR ‘Nurse’) OR (‘ambulance’ OR ‘Emergency Medical Service’ or ‘EMS’ OR ‘pre Hospital’ OR ‘Emergency Department’). Limitations will be applied to the search to return results in the English language, but no limits on publication dates are included.

### Deciding what is relevant to the initial interest

We anticipate that due to the limited literature pertaining to paramedics, all articles with a paramedic focus on heroism will be selected for review and manually scanned for relevance and eligibility. We will use the following criteria to select studies for analysis:

1. The expressed a priori purpose of the study was to examine paramedic, nurse or doctor
2. A focus on heroism or hero/heroine or for paramedics
3. Ambulance OR emergency OR pre-hospital care OR EMS
4. The studies were conducted using qualitative methods OR Narrative OR Opinion pieces.

### Reading studies and extracting data

We will conduct and report the metasynthesis in accordance with the Preferred Reporting Items for Systematic review and Meta-Analysis Protocols (PRISMA-P) checklist (Shamseer et al., 2015). In order to eliminate publications that are clearly not within the inclusion criteria, a review of each citation title and abstract will be conducted between two reviewers to identify articles likely to be eligible. Any disagreements between reviewers will be resolved through discussion or by a third reviewer.

### Assessment of quality of included studies

The two reviewers will independently apply the Critical Appraisal Skills Programme (CASP) quality assessment tool for qualitative studies (Atkins et al., 2008) as adapted by Ochodo et al. (2017), which has been applied in previous reviews of qualitative studies (Ames & Lewin, 2015; Glenton et al., 2013). Disagreements will again be resolved though discussion or by a third reviewer. This checklist will have the following questions scored as either Yes, No or Unclear:

1. Is the study setting or context described sufficiently?
2. Is the sampling method clearly described?
3. Is the data collection method clearly described?
4. Is the method for data analysis clearly described and appropriate?
5. Are the findings or conclusions made supported by adequate evidence?
6. Is there evidence of reflexivity?
7. Does this study demonstrate sensitivity to ethical concerns?
8. Any other concerns?

### Determining how studies are related

This will involve open coding where each article will be read; findings will be highlighted and compared for similarities, differences and questions regarding emergent phenomena. This will involve identification of indicators which are words or phrases of interest.

### Translating studies

**Figure 1:**
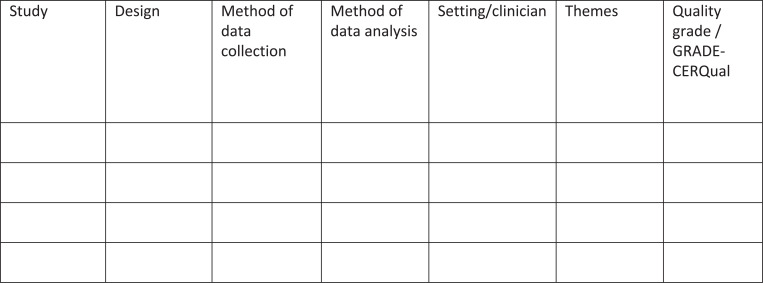
Figure 1. Data abstraction form.

Study manuscripts will be extracted into NVIVO V.12. A data abstraction form (Figure 1) will be completed for each study and will capture the study authors, design, method of data collection, method of data analysis, setting/clinician, themes and quality.

We will use the concept-indicator model advocated by Strauss (1987). This model has previously been successfully used by members of the study team with a metasynthesis on paramedic practice and is described in Rees et al. (2015). Each article will be read, and the findings will be coded in NVIVO V.12 and compared for similarities, differences and questions regarding emergent phenomena. Indicators are sentences, phrases or words, while concepts are labels associated with indicators. Through constant comparisons within the texts, indicators will be grouped under higher conceptual headings, known as categories, and when an indicator does not generate new insights in concepts, these categories will be deemed theoretically saturated and well grounded.

### Assessment of confidence in results

Two of the review authors will independently assess the quality of studies using the approach of Grading of Recommendations Assessment, Development and Evaluation-Confidence in Evidence from Reviews of Qualitative research (GRADE-CERQual) (Lewin et al., 2018). CERQual involves an assessment of each individual review finding in terms of four components:

1. Methodological limitations
2. Coherence
3. Adequacy of data
4. Relevance.

The overall confidence in each review finding (i.e. for each theme generated) will be graded independently by two reviewers as: high, moderate, low or very low GRADE-CERQual. Disagreements between the reviewers will once again be discussed until consensus is achieved.

### Synthesising translations

This will involve selective coding which entails ‘explication of the story line’ (Strauss & Corbin 1998, p. 148). We will return to the wider body of literature in order to conceptualise and synthesise studies within the wider body of knowledge and across contexts.

### Expressing the synthesis

The final stage of coding will involve weaving the theoretically saturated categories together with the body of knowledge and constructing a narrative on heroism and paramedic practice.

### Reporting of protocol and metasynthesis

**Figure 2:**
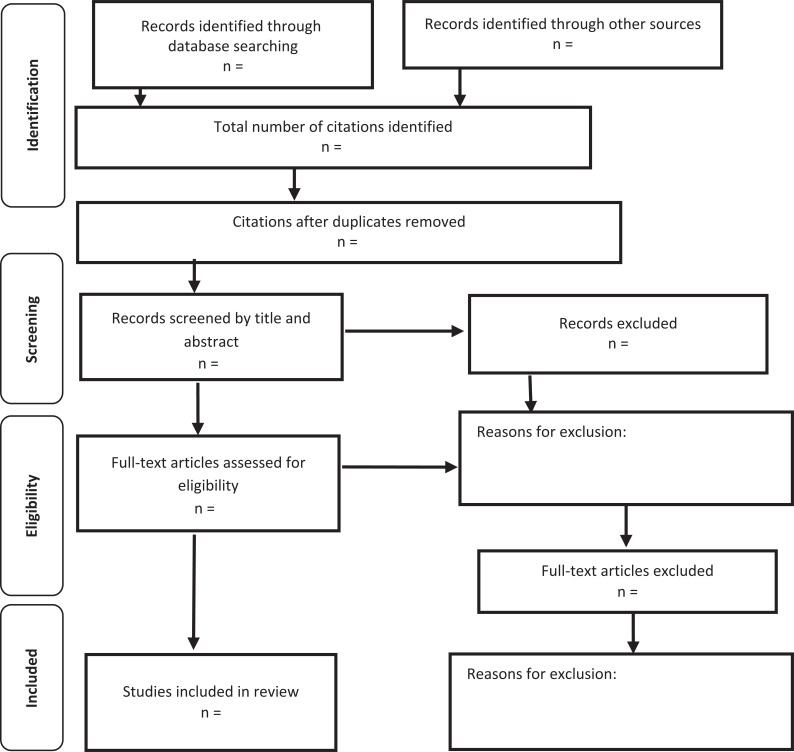
Figure 2. Based on Moher et al. (2009).

The trustworthiness of this metasynthesis is established through transparent data collection, extraction and analysis methods (Finfgeld, 2003). PRISMA-P was adopted when preparing this protocol. As outlined within these reporting guidelines, we will also use PRISMA (Moher et al., 2009; Figure 2) to report the findings of our metasynthesis, as PRISMA focuses on reporting of intervention reviews. We are tailoring this reporting to reflect the qualitative nature of this metasynthesis.

## Conclusion

Narratives on heroism and paramedic practice are important in terms of the relationship between paramedics and society. The metasynthesis prospectively reported in this article serves as the first point in our journey of making sense of and developing a theory on heroism and paramedic practice.

## Author contributions

NR led on preparing the manuscript. LS, TA and JW collaborated on the design, and all approved the final manuscript. NR acts as the guarantor for this article.

## Conflict of interest

None declared.

## Disclaimer

The views and opinions expressed herein are those of the authors and do not necessarily reflect those of WAST.

## Ethics

The protocol has been registered with the International Prospective Register of Systematic Reviews PROSPERO 2021, registration number CRD42021234851. We will also prospectively disseminate our protocol publicly through publication in a peer-reviewed journal and via conference presentations. This metasynthesis is a retrospective study drawing on publicly available data; it does not involve NHS staff or patients and so NHS Research Ethical review was not required within Health Research Authority guidance (HRA, 2020).

## Funding

This project is supported by WAST R&D investigator funding.
